# Supplementary material for: Raman Spectroscopy Profiling of Splenic T-Cells in Sepsis and Endotoxemia in Mice
Source: Int J Mol Sci. 2023 Jul 27;24(15):12027. doi: 10.3390/ijms241512027 (PMC10419286; doi:10.3390/ijms241512027)
Supplement: Supplementary file 1 [file ijms-24-12027-s001.zip › ijms-2424258-supplementary.pdf]

Raman Spectroscopy Profiling of Splenic T-Cells in Sepsis and Endotoxemia in Mice

Ibukun Elizabeth Osadare [1], Ling Xiong [2], Ignacio Rubio [2,3,4], Ute Neugebauer [1,2,5], Adrian T. Press [2,3,4,6], Anuradha Ramoji [1,5,*] and Juergen Popp [1,5]

1 Institute of Physical Chemistry (IPC), Abbe Center of Photonics (ACP), Friedrich Schiller University Jena, Member of the Leibniz Centre for Photonics in Infection Research (LPI), Helmholtzweg 4, 07743 Jena, Germany; ibukun.osadare@uni-jena.de (I.E.O.); ute.neugebauer@med.uni-jena.de (U.N.); juergen.popp@ipht-jena.de (J.P.)

2 Department of Anesthesiology and Intensive Care, Jena University Hospital, Friedrich Schiller University Jena, Am Klinikum 1, 07747 Jena, Germany; ling.xiong@med.uni-jena.de (L.X.); ignacio.rubio@med.uni-jena.de (I.R.); adrian.press@med.uni-jena.de (A.T.P.)

3 Center for Sepsis Control and Care (CSCC), Jena University Hospital, Friedrich Schiller University Jena, Am Klinikum 1, 07747 Jena, Germany

4 Leibniz Center for Photonics in Infection Research, Jena University Hospital, Friedrich Schiller University Jena, 07747 Jena, Germany

5 Leibniz Institute of Photonic Technology, Member of Leibniz Health Technologies, Member of the Leibniz Centre for Photonics in Infection Research (LPI), Albert-Einstein-Straße 9, 07745 Jena, Germany

6 Faculty of Medicine, Friedrich Schiller University Jena, Kastanienstraße. 1, 07747 Jena, Germany

* Correspondence: anuradha.ramoji@uni-jena.de; Tel.: +49-3641-9390926

Supplement Figures

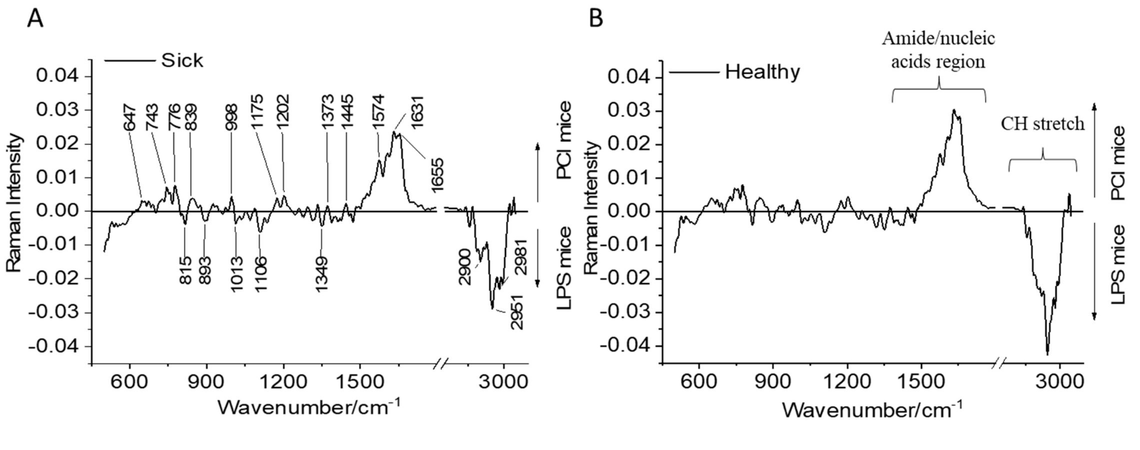

Figure S1: Raman difference spectra generated by subtracting C) mean Raman spectra of T-lymphocytes isolated from the spleen of endotoxin mice from septic mice. D) For comparison, the Raman difference spectra were generated by subtracting the mean Raman spectra of T-lymphocytes isolated from the spleen of healthy mice within the endotoxemia model and the PCI models.

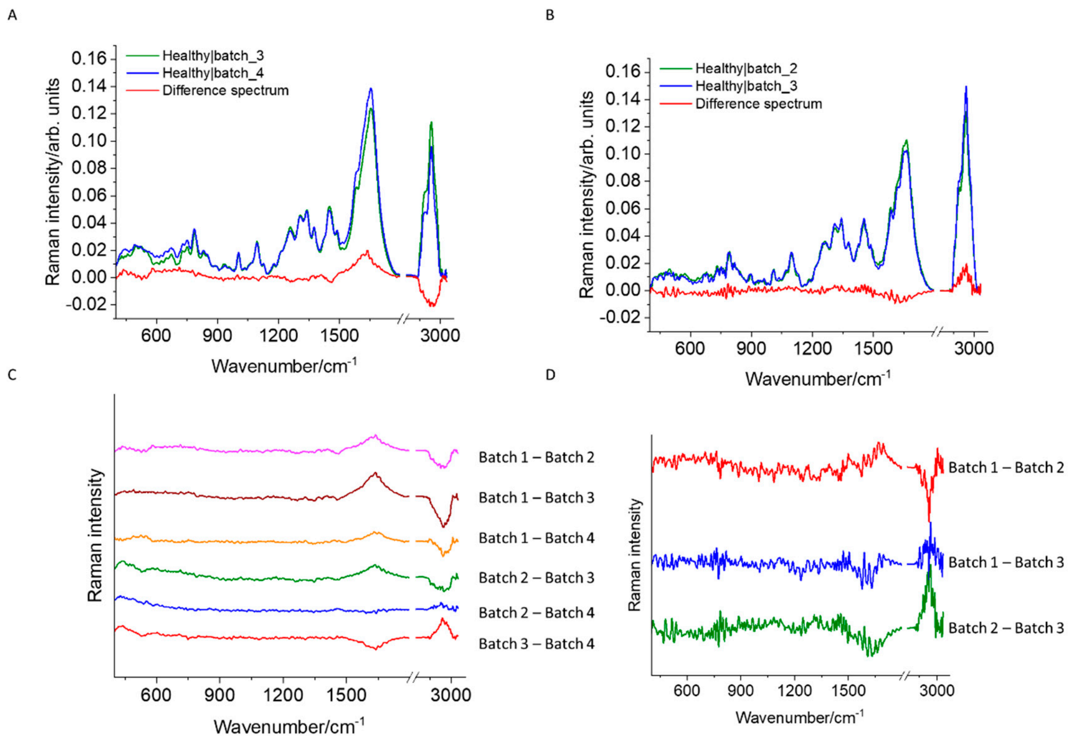

Figure S2: Raman difference spectra generated by subtracting mean Raman spectra of A) control mice from experimental batch 3 and batch 4 of the PCI model, B) control mice from experimental batch 2 and batch 3 of the endotoxemic model. Raman difference spectra for C) PCI model control mice from different experimental batches, D) endotoxemic model control mice from different experimental batches.

Table S1: A) Experimental details of T-cells isolated from the spleen of control mice, septic mice (PCI), and endotoxemic mice (LPS) showing the number of biological replicates, and gender of mice.

**Mice with LPS insult**			
**Biological replicates**	**9 to 12 mice/ batch**	**Post-LPS insult time**	**Gender of mice**
Batch 1	Pooled	One day	Females and Males
Batch 2	Pooled	One day	Females and Males

Batch 3	Pooled	One day	Females and Males
**Mice with PCI insult**			
**Biological replicates**	**Mice/batch**	**Post PCI insult time**	**Gender of mice**
**Batch 1**			
PCI_73646<br>PCI_73647<br>Sham_73670<br>Sham_73671	4 mice (2 control and 2 septic)	One day	Females
**Batch 2**			
PCI_73648<br>PCI_73666<br>Sham_73678<br>Sham_73679	4 mice (2 control and 2 septic)	One day	Females<br>Males<br>Males<br>Males
**Batch 3**			
PCI_73667<br>PCI_73668<br>Sham_73677<br>Sham_73680	4 mice (2 control and 2 septic)	One day	All males
**Batch 4**			
PCI_73669<br>PCI_73681<br>Sham_73682<br>Sham_73683	4 mice (2 control and 2 septic)	One day	All males
**Batch 5**			
PCI_73739<br>PCI_73740	2 septic mice	One day	All females

Table S2: Batch-wise confusion matrix of Raman models for differentiation of T-lymphocytes from sham and septic/inflammation mice.

CLASSIFICATION MODELS using 10-fold cross-validation	**Balanced accuracy*/Cohen's kappa**					
	PCI mice model			LPS mice model		
	PCA-LDA	PCA-RF	PCA-SVM	PCA-LDA	PCA-RF	PCA-SVM
BATCH 1	0.85/0.71	0.86/0.72	0.85/0.71	0.60/0.19	0.60/0.19	0.62/0.23
BATCH 2	0.67/0.33	0.76/0.52	0.77/0.53	0.82/0.64	0.83/0.66	0.87/0.74
BATCH 3	0.72/0.43	0.76/0.52	0.81/0.60	0.71/0.42	0.71/0.42	0.72/0.44
BATCH 4	0.84/0.67	0.83/0.66	0.82/0.63	-	-	-
*Balanced accuracy is the mean sensitivity						

Table S3: Confusion matrix obtained from PCA-LDA classification model built to differentiate T-lymphocytes from sham and infected mice from PCI model.

PCA-LDA classification model (batches 2 to 5) for PCI using batch-wise cross-validation								
	Confusion matrix for individual aggregated spectra				Majority vote			
True/Predicted	Sham	Infected	Sens	Spec	Sham	Infected	Sens	Spec
Sham	356	264	0.57	0.75	1	0	1	1
Infected	174	519	0.75	0.57	0	1	1	1
Balanced accuracy* /Cohen's kappa	0.67/0.33				1/1			
*Balanced accuracy is mean sensitivity, Sens: sensitivity, Spec: specificity								

Table S4: Confusion matrix obtained from PCA-LDA prediction of T-lymphocytes from septic mice.

PCA-LDA prediction model (batch 1) for PCI								
	Confusion matrix for individual aggregated spectra				Majority vote			
True/Predicted	Sham	Infected	Sens	Spec	Sham	Infected	Sens	Spec
Sham	134	49	0.73	0.85	1	0	1	1
Infected	23	130	0.85	0.73	0	1	1	1
Balanced accuracy* /Cohen's kappa	0.79/0.57				1/1			
*Balanced accuracy is mean sensitivity, Sens: sensitivity, Spec: specificity								

Table S5: Confusion matrix obtained from PCA-LDA classification model built to differentiate T-lymphocytes from sham and infected mice from LPS model.

PCA-LDA classification model (batches 1 and 3) for LPS using batch-wise cross-validation								
	Confusion matrix for individual aggregated spectra				Majority vote			
True/Predicted	Sham	Infected	Sens	Spec	Sham	Infected	Sens	Spec
Sham	1535	33	0.98	0.04	1	0	1	0
Infected	1398	51	0.04	0.98	1	0	0	1
Balanced accuracy* /Cohen's kappa	0.51/0.02				0.50/0			
*Balanced accuracy is mean sensitivity, Sens: sensitivity, Spec: specificity								

Table S6: Confusion matrix obtained from PCA-LDA prediction of T-lymphocytes from LPS mice.

PCA-LDA prediction model (batch 2) for LPS								
	Confusion matrix for individual aggregated spectra				Majority vote			
True/Predicted	Sham	Infected	Sens	Spec	Sham	Infected	Sens	Spec
Sham	78	595	0.12	0.68	0	1	0	1
Infected	230	497	0.68	0.12	0	1	1	0
Balanced accuracy* /Cohen's kappa	0.4/-0.21				0.50/0			
*Balanced accuracy is mean sensitivity, Sens: sensitivity, Spec: specificity								
